# Codesigning a Digital Type 2 Diabetes Risk Communication Tool in Singapore: Qualitative Participatory Action Research Approach

**DOI:** 10.2196/50456

**Published:** 2024-11-05

**Authors:** Jumana Hashim, Lidia Luna Puerta, Pin Sym Foong, E Shyong Tai, Huso Yi, Helen Elizabeth Smith

**Affiliations:** 1 Saw Swee Hock School of Public Health National University of Singapore Singapore Singapore; 2 Lee Kong Chian School of Medicine Nanyang Technological University Singapore Singapore Singapore; 3 Telehealth Core National University of Singapore Singapore Singapore; 4 Division of Endocrinology National University Health System Singapore Singapore

**Keywords:** type 2 diabetes, risk perception, co-design, risk communication tool, diabetes prevention

## Abstract

**Background:**

Diabetes is a serious public health concern worldwide. Despite public health efforts encouraging early screening and improving knowledge of effective interventions for those at increased risk of type 2 diabetes (T2D), the incorporation of preventative behaviors into an individual’s daily life remains suboptimal. Successfully and accurately increasing risk perception has been demonstrated to increase behavioral intention.

**Objective:**

The study aims to codesign a T2D risk communication tool by engaging public participants to (1) identify key characteristics that contribute to an effective risk communication tool and (2) test and iterate to develop a culturally sensitive and meaningful risk communication tool that can motivate T2D preventative behaviors.

**Methods:**

We adopted a novel methodology, “Patient and Public Involvement (PPI) Hawkers,” where we approached patrons at hawker centers and public eateries frequented by all local residents to evaluate and test 3 prototypes for the tool. The three prototypes were (1) “Diabetes Onset”—estimated age of diabetes onset of T2D based on one’s risk factors, (2) “Relative Risk”—the relative risk of T2D is presented in a 1-10 scale indicating where one’s risk score lie in relation to others, and (3) “Metabolic Age”—the median age of the risk category based on one’s risk factors, presented to be compared against their chronological age. We gathered reactions and feedback through rapid testing and iteration to understand which risk result presentation would be received the best. All the collected data were revisited and analyzed using an inductive thematic analysis to identify the key characteristics contributing to an effective risk communication tool.

**Results:**

We engaged with 112 participants (female: n=59, 56%) across 6 hawker centers. The key characteristics that were important to participants emerged in four main themes: (1) appeal and user experience, in terms of format and readability; (2) trust and validity of the institution providing the tool and the accuracy of the risk result; (3) threat appraisal: salience of risk information, which influenced their risk perception; and (4) coping appraisal: facilitators for behavior change, which impacted their intention for implementing T2D preventative behaviors. The predictive nature of the prototype entitled “Diabetes Onset” was poorly received and removed after the first iteration. The Relative Risk prototype was valued for being straightforward but feared to be boring. The Metabolic Age prototype was anticipated to be more motivating for behavior change, but there were some concerns that the terminology may not be understood by everyone.

**Conclusions:**

Participants were divided on which of the 2 prototypes, “Metabolic Age” or “Relative Risk,” they would favor adopting. Further testing is now required to determine which prototype will be more effective in motivating behavior change. This study’s insights on the design process and valued characteristics of a risk communication tool will inform future development of such interventions.

## Introduction

Accelerating rates of diabetes incidence have given rise to a global public health epidemic. Diabetes imposes a large burden of morbidity and mortality, as well as an economic burden, on society. Lifestyle changes, such as weight management, physical activity, and healthy eating, can reduce the risk of developing type 2 diabetes (T2D) by up to 53% [1]. Singapore’s government has recognized the magnitude of T2D as a public health problem, declaring a “War on Diabetes.” Screening has been prioritized, and resources have been allocated to promote physical activity, including the “National Steps Challenge.” In a Singapore National Health Survey, 70% of respondents who were unaffected by diabetes reported having gone for screening within the recommended time, and 87% strongly agreed that exercise and healthy eating can control the risk of diabetes [2]. However, the uptake of recommended behavior change remains limited; a 2019 study found that only 28% of Singaporeans met the Health Promotion Board’s physical activity recommendation of 150 minutes per week, and only 37% ate 5 daily servings of fruits and vegetables [3]. This illustrates the tenuous link between knowledge of disease and the adoption of preventative behaviors.

Effective risk communication is essential in public understanding of their health status and promoting positive behavior change [4,5]. Successfully and accurately increasing risk perception has been demonstrated to increase behavioral intention [6]. However, presenting risk information alone has small effects on cognitive processes for behavior adoption unless actionable information that enhances autonomy and self-efficacy is also given [7,8]. Protection Motivation Theory incorporates these 2 elements and suggests that 2 pathways influence the subsequent intention of practicing health-promoting behaviors: threat and coping appraisals [9]. Threat appraisal considers the risk perception of T2D through perceived severity and perceived vulnerability. Coping appraisal considers if the recommended behavior is actionable and efficient given the perceived costs and benefits. Our previous qualitative work in exploring lay perceptions of T2D suggests that both the perceived threat of T2D and the coping appraisal related to prevention did not provide sufficient motivation to undertake lifestyle changes [10]. Perceived threat is low as complications of T2D, such as limb amputations and blindness, were seen downstream of T2D onset and can be prevented with management of T2D after diagnosis. The centrality of food in Singapore’s culture also resulted in a high perceived response cost, resulting in a negative impact on coping appraisal. Messaging to inform individual risk and promote preventative measures are needed to influence these gaps in threat and coping appraisal accordingly.

One way to identify individuals at increased risk of diabetes is to measure blood sugar or hemoglobin A_1c_ (HbA_1c_; an estimate of mean glucose). The population is often dichotomously classified as having prediabetes if they have levels of these parameters above a threshold of 5.7% HbA_1c_. This approach is problematic in 2 ways. First, dichotomous messaging that is used in Singapore and in many other places may give rise to a false sense of security in those who fall just below the threshold. Given that the risk of T2D is continuous, we may miss opportunities to motivate behavioral change in those at lower, but nevertheless elevated, risk of T2D and who would benefit from lifestyle change. Second, even though diabetes is characterized by elevated blood glucose, T2D is a multidimensional disease, and the current approach has been criticized for being overly gluco-centric [11].

Risk prediction based on multiple variables, on top of blood glucose, has shown to be a better estimate of risk. Recent developments to include additional clinical parameters like body mass index, systolic blood pressure, triglyceride, and high-density lipoprotein cholesterol have shown to have good accuracy in predicting the risk of developing T2D [12]. Adopting such a strategy can move away from being gluco-centric and shift preventative efforts upstream instead of waiting for individuals to become prediabetic. However, the interpretation of the output from these multivariate predictive functions can be challenging. Communicating risk through percentage risk over the next 10 years (absolute risk) has shown to be falsely reassuring as the absolute risk tends to be numerically quite small. Generally, when faced with scales (like 0-100), people do not find percentages below 50 concerning [3,13]. Visual imagery or analogies are examples of relevant and meaningful risk presentations that can increase the intention for behavioral change [14-16]. Following risk information, demonstration of how the risk can be reduced is critical as the components of coping appraisal have shown to have the strongest predictors of practiced behavior change [17-19].

We sought to design and develop a novel risk communication tool to enhance threat appraisal while positively influencing coping appraisal. Leveraging participatory action research, we engaged and involved members of the public to identify feasible risk communication tools so that they are customized for the prevention of T2D in Singapore. To support future scalability as well as to allow the risk communication tool to be dynamic and interactive, the tool was intended to be delivered digitally, likely as a website to promote ease of access. The study objectives were to (1) identify key characteristics that contribute to an effective risk communication tool and (2) test and iterate to develop a culturally sensitive and meaningful risk communication tool that can motivate T2D preventative behaviors.

## Methods

### Developing the Risk Communication Prototypes

We facilitated 2 ideation workshops to codesign ways to present risk information. These workshops adopted a design-thinking approach and included members of the public, health care professionals with experience in diabetes, and digital tool designers. First, participants were guided to define potential problems that could arise from identified gaps in the threat and coping appraisal of T2D prevention. Second, participants were asked to ideate on messaging solutions to address the problems they identified.

The study team took the messaging solutions discussed in the workshops to create potential prototypes using message-framing strategies described in the Tripartite Model of Risk Perception (TRIRISK), heuristics from behavioral economics theories, and existing risk communication literature [7,19-21]. At this stage of the development, the prototypes were nonfunctional as the focus was on the risk messaging and design. However, eventually, the aim is to incorporate the multi-factorial risk prediction model [12] and convert it into the framing and/or analogy based on the messaging concept that tests to be the best received. Hence, to ensure these messaging concepts were realistic, we checked the feasibility and validity of data with the experts who built the multifactorial risk prediction model. This left us with 3 designs for risk result presentation prototypes:

1. “Diabetes Onset”: estimated age of diabetes onset of T2D based on one’s risk factors was designed as a fear appeal to elevate threat appraisal. The higher the elevated risk factors, the sooner the estimated age of diabetes onset. This leverages the concept of rate advancement periods [22], which translates the impact of certain risk factors in terms of time of chronic disease occurrence.
2. “Relative Risk”: the relative risk of T2D was presented using a 1-10 scale indicating where one’s risk score was in relation to others. This translated absolute risk to standardized risk percentiles [23], making it more relevant and appropriately positioned to understand one’s risk. To demonstrate that this risk increases over time with no action, the relative risks for now, in 5 years, and in 10 years’ time were presented.
3. “Metabolic Age”: the median age of the risk category based on one’s risk factors, presented to be compared against their chronological age. Metabolic age was reflected as being older the more the risk factors were elevated; this is a form of relative risk measurement but presented in a different way. Risk communicated through “age” tools has shown to be effective in changing patient behavior given the strong desire for delayed aging and continued youthfulness [24,25].

The risk functions were not integrated in this phase of development, so the prototypes presented a dummy risk results page according to the 3 designs outlined above. Each prototype also had an introduction, a data input page, and an intervention page. The introduction page was designed to address the constructs of threat appraisal by addressing perceived vulnerability, as 1 in 3 Singaporeans are diagnosed with T2D. The data page was prefilled with the parameters required for the multifactorial risk model (eg, age, BMI, parental history, hypertension, triglycerides, and HbA_1c_). The intervention page provided the opportunity for users to observe the impact of preventative action on their risk of T2D to increase self-efficacy and autonomy, facilitating their coping appraisal.

### Study Design

To gather a diverse and wide set of input for our objectives, we used the “Patient and Public Involvement Hawker” (PPI Hawker) method [26]. Hawker centers are open, noninstitutional, and public space food stalls where a large proportion of the Singapore population purchase their meals. The unstructured, short, and informal nature of this method facilitates engagement with those who do not usually participate in health research.

To plan and conduct the “PPI Hawker” sessions, we followed the step-by-step guide described in the original publication [26]. The study team was assisted by 4 lay facilitators. These facilitators were recruited from the ideation workshops and snowballing approach. The recruitment strategy for facilitators was driven by ensuring that at least one of the facilitators could speak each of the local languages: Mandarin, Malay, and Tamil, in addition to English.

To interact with participants, we went to 6 hawker centers across Singapore. We approached hawker center patrons who appeared to be between 30 and 60 years of age. Purposive sampling was used to engage groups of the population not involved in previous discussions to ensure demographic diversity. For example, if we had interacted with mostly females of Chinese ethnicity, we would try to approach males and other ethnicities to diversify the perspectives we captured. Upon approaching potential participants, the facilitators would briefly introduce themselves and the study and ask if they would be willing to engage in a 5- to 10-minute discussion. If the participants give verbal consent, facilitators will provide more context and pose the questions. To avoid overwhelming participants and to moderate the time needed, we began by showing only 1 of the 3 paper-based prototypes during each interaction. Once the prototypes and prompts became more refined, in the latter sessions, we were able to show 2 prototypes during each interaction for comparison.

### Data Collection

The lay facilitators refined the discussion prompts produced by the study team to reduce jargon and increase relatability. The initial discussion prompts were guided by 4 categories of inquiry: comprehensibility, relatability, usability, and impactfulness. These prompts evolved throughout the study and as the prototypes were iterated.

Since no identifiable data was collected, the perceived age and ethnicity of each participant were noted. We began by presenting an A3-sized paper-based prototype to participants during each interaction. In the latter sessions, we presented the clickable prototypes on an iPad for participants to interact with. An A4 copy of the prototype presented in each encounter was used by a study team member to note feedback and suggestions to allow circling and annotation of the different features participants may be referring to. At the end of each session, notes and insights were discussed with the lay facilitators to ensure that all the comments and feedback were accurately captured and whether we had reached thematic saturation and sample diversity for that particular iteration. Any discrepancies in understanding the data collected or contradictory insights among interactions were discussed and noted accordingly.

### Prototype Iteration and Analysis

After each hawker session, notes were consolidated and summarized for each prototype. If consensus were reached regarding thematic saturation and sample diversity. The next session was scheduled once the prototypes and prompts were revised according to the identified insights. We revised the prototypes and prompts twice, which produced 3 iterations (sessions 1 and 2 as the first iteration, sessions 3 and 4 as the second iteration, and sessions 5 and 6 as the third iteration). At the end of all the sessions, JH and LLP revisited all the data collected using an inductive thematic analysis to identify the key characteristics that contributed to an effective risk communication tool and shaped the various prototype iterations.

### Ethical Considerations

This study received approval from the Nanyang Technological University institutional review board (approval number IRB-2021-01-041). A waiver for written consent was approved; hence, verbal consent was obtained from each participant before each interaction. No identifiable information was collected, and each interaction was anonymously annotated using an arbitrary participant number. After each interaction, the facilitators offered to buy a beverage for the participant as a token of appreciation.

## Results

Across the 6 hawker centers, we engaged with 112 participants, where 59 (56%) were perceived to be female. In total, 50 (45%) participants were identified as Chinese, 33 (29%) as Malay, and 25 (22%) as Asian Indian. Table 1 contains the breakdown of participants across the different iterations.

**Table 1:** Location of each hawker session and perceived demographic information of participants who agreed to engage in a discussion with the facilitators.

			Iteration 1 (n=36)		Iteration 2 (n=42)		Iteration 3 (n=34)	
**Hawker center sessions**								
	Location A	Ghim Moh Market & Food Centre		Whampoa Food Centre		Ayer Rajah Food Centre		
	Location B	Upper Boon Keng Market & Food Centre		Boon Lay Place Market and Food Village		Tekka Centre		
**Sex, n**								
	Male	16		27		16		
	Female	20		15		18		
**Ethnicity, n**								
	Chinese	25		15		10		
	Malay	4		14		15		
	Tamil	3		13		9		
	Other	4		0		0		

**Figure 1:**
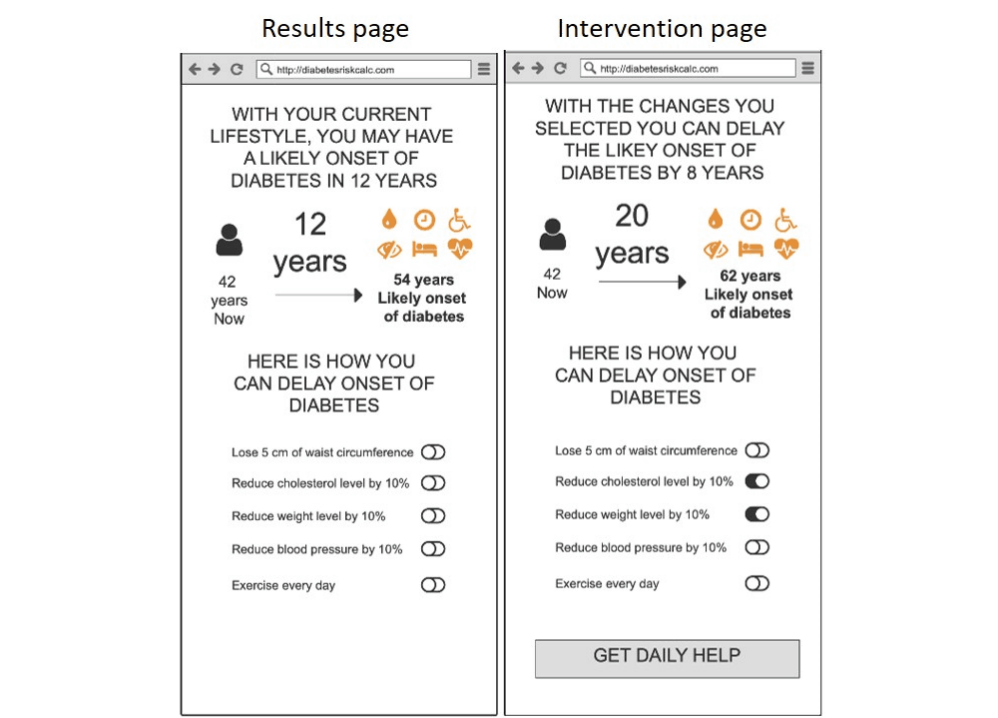
Wireframe prototype of “Diabetes Onset” used in Iteration 1, printed on A3 paper, to gather feedback from participants in a “PPI Hawker” study.

**Figure 2:**
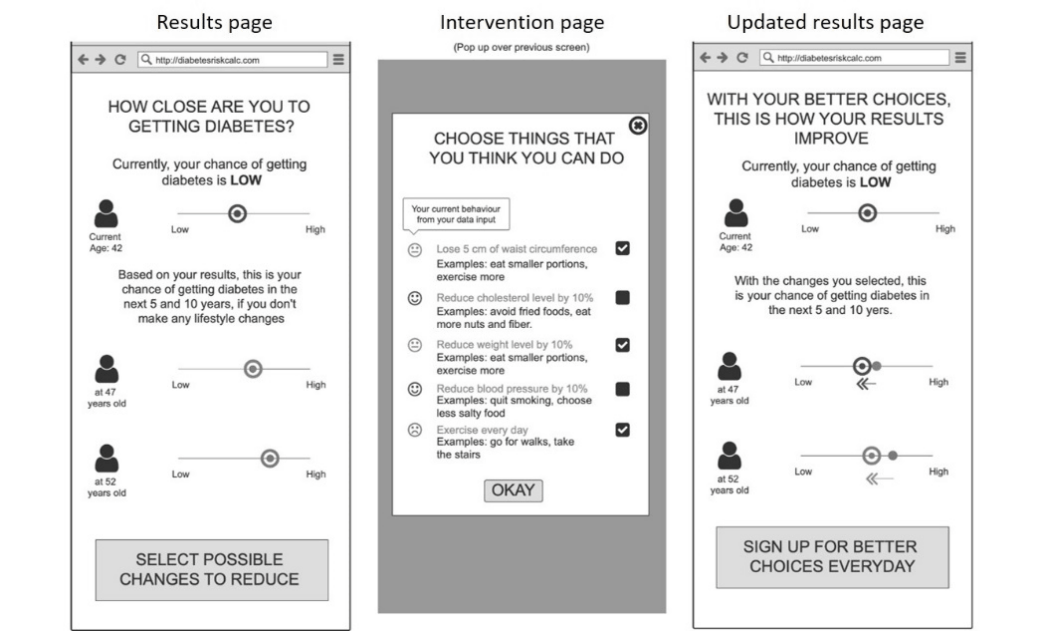
Wireframe prototype of “Relative Risk” used in Iteration 1, printed on A3 paper, to gather feedback from participants in a “PPI Hawker” study.

**Figure 3:**
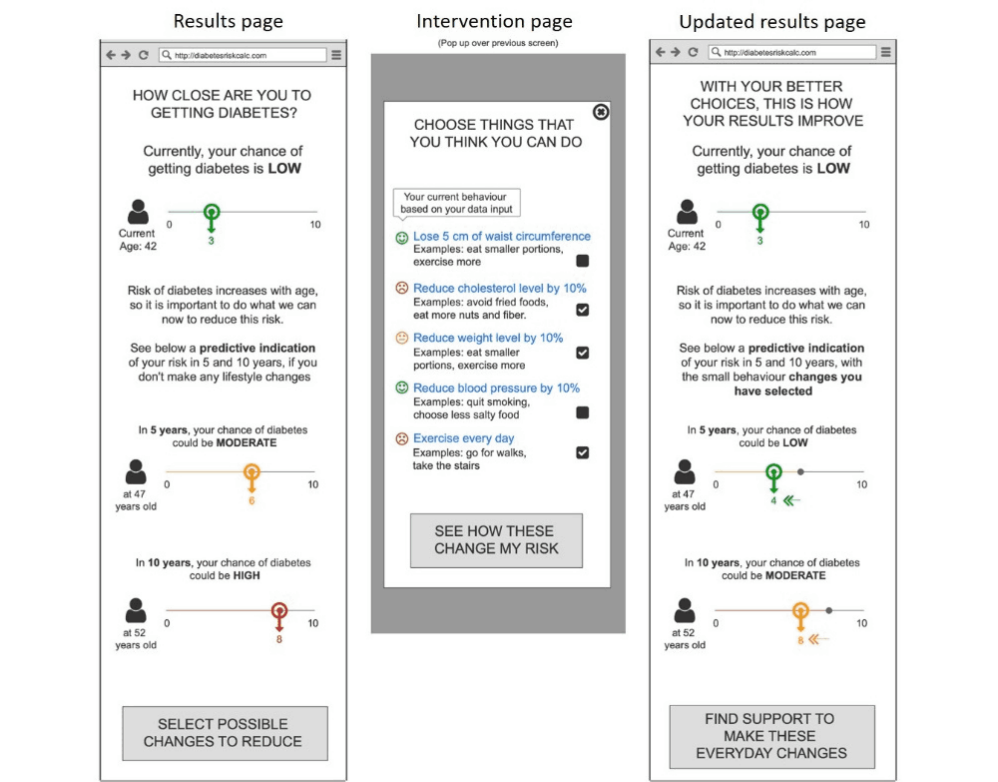
Wireframe prototype of “Relative Risk” used in Iteration 2, printed on A3 paper, to gather feedback from participants in a “PPI Hawker” study.

**Figure 4:**
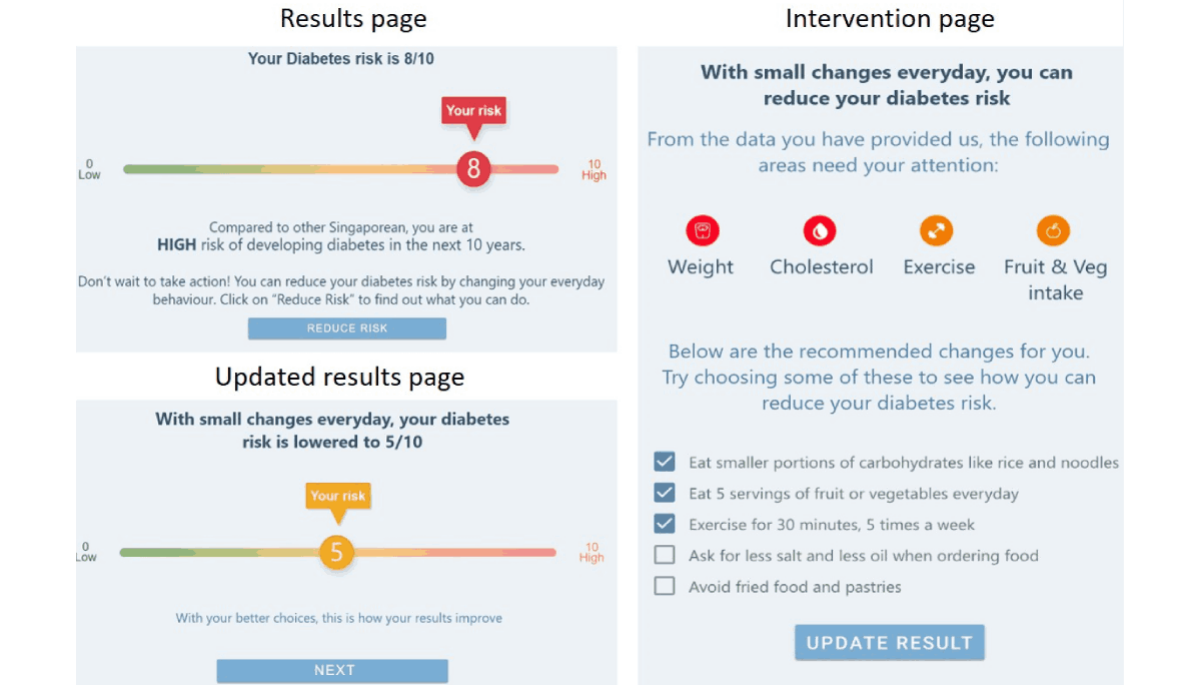
Clickable Prototype of “Relative Risk” used in Iteration 3, on iPad, to gather feedback from participants in a “PPI Hawker” study.

**Figure 5:**
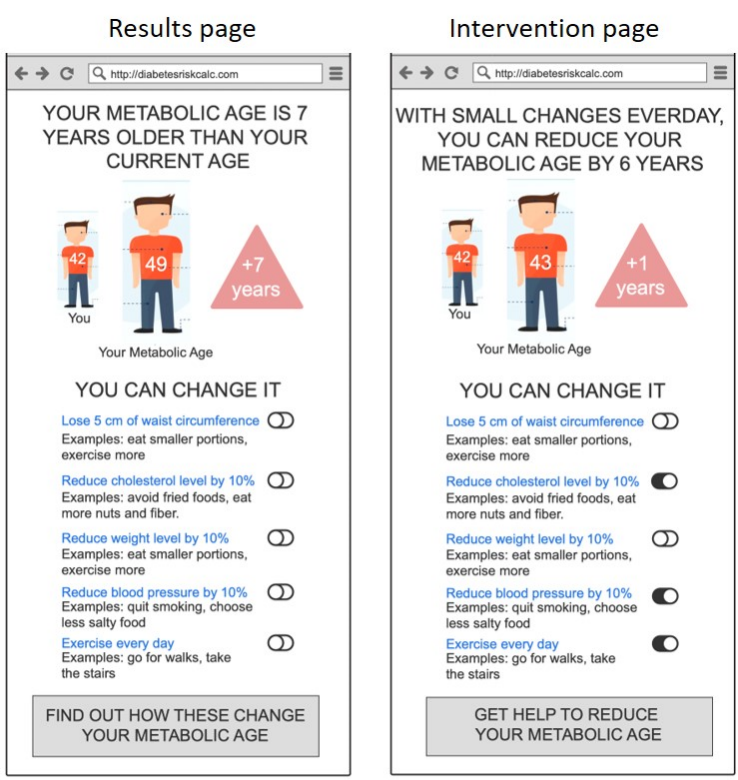
Wireframe prototype of “Metabolic Age” used in Iteration 1, printed on A3 paper, to gather feedback from participants in a “PPI Hawker” study.

**Figure 6:**
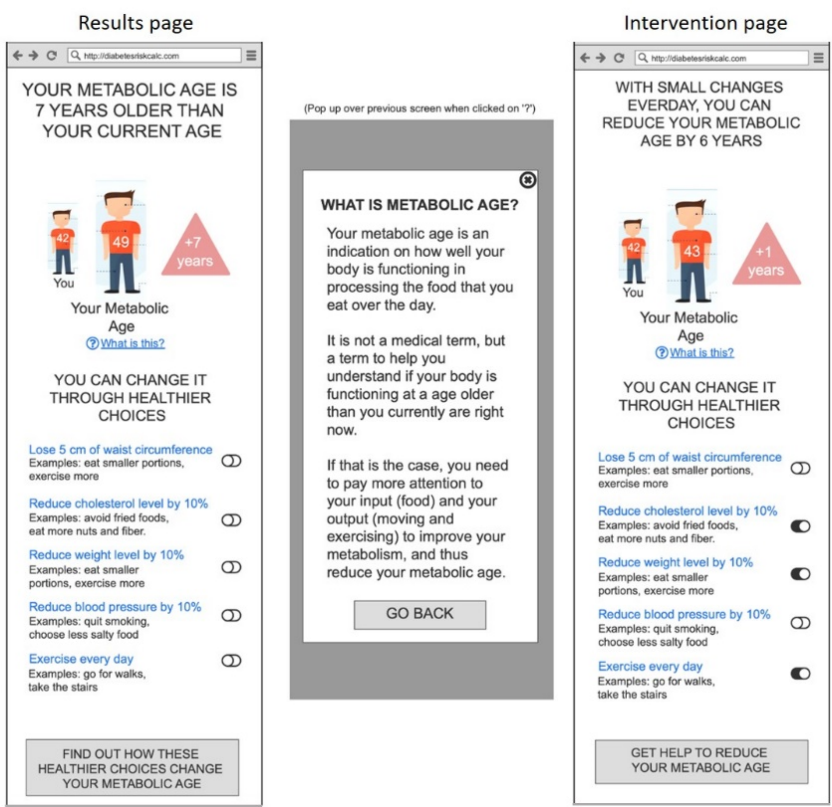
Wireframe prototype of “Metabolic Age” used in Iteration 2, printed on A3 paper, to gather feedback from participants in a “PPI Hawker” study.

**Figure 7:**
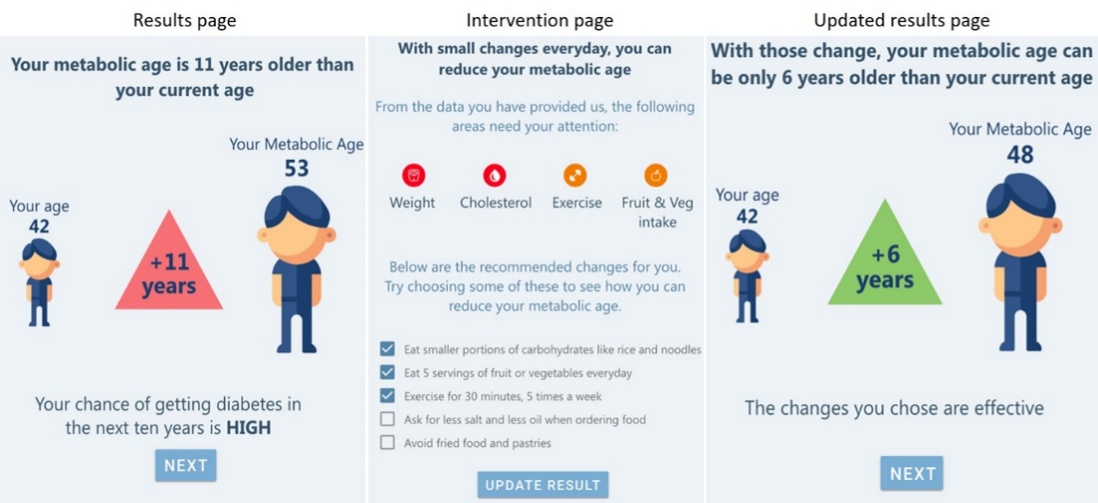
Clickable prototype of “Metabolic Age” used in Iteration 3, on iPad, to gather feedback from participants in a “PPI Hawker” study.

The key characteristics that shaped the iterations emerged as four main themes: (1) appeal and user experience, (2) trust and validity, (3) threat appraisal: salience of risk information, and (4) coping appraisal: facilitators for behavior change. Based on these findings, we were able to do rapid iterations to refine the prototypes. The different iterations of the prototypes are demonstrated in Figures 1-7.

### Appeal and User Experience

Comments on first impressions across all the prototypes are often related to readability and appeal, especially in the first iteration. All participants expressed the need for more concise and accessible text and the inclusion of visual and interactive elements to increase sophistication and enhance the appeal of the risk communication prototypes. With the introduction of color in the second iteration, participants commented spontaneously on how their understanding of risk was intuitive.

The risk communication tool’s availability only in English, rather than all 4 of the official languages of Singapore, was perceived as a limit to accessibility. Some participants experienced difficulty reading and suggested audio, video, or diagrams. There was a consensus that typing should be minimized and replaced with drop-down lists, checkboxes, or sliders.

For the third iteration, clickable prototypes were presented on an iPad for participants to have a realistic experience of the risk communication tool. We observed that people interact with digital media copies and print copies differently. Participants rapidly clicked and scrolled through without taking the time to read each screen, whereas in the print copies, participants went through each section more carefully.

### Trust and Validity

Many participants suggested the public’s perception of trustworthiness in risk communication would be increased if health care providers promoted the use of the tool. Furthermore, some noted it may help to track their risk over time and to incentivize them to maintain healthy behavior between appointments or screenings.

Some participants noted that the requirement to input health screening information (ie, blood pressure, triglyceride, HbA_1c_, etc) instead of just self-reported survey questions made the risk score generated more “real.” A participant positively referred to it as “based on the body and not just guessing from how many sugary drinks [someone] had.” Some suggested data integration between existing electronic medical records and the tool to observe risk trends over time.

At the end of the first iteration, there was an overwhelming consensus that the “Diabetes Onset” prototype was too negative and inaccurate. Their perception was that predictions of when one would get diabetes were not grounded in evidence and made them distrustful of the tool. One participant questioned how we could know when he will get diabetes and asked, “Do you have a crystal ball?” Hence, we did not test this prototype in further iterations.

The credibility of the institution providing the tool was important to participants. They shared preferences for the tool to be managed by governmental bodies or known health care institutions. They noted that the tool would need to include information on how information entered would be managed, protected, and stored. Participants emphasized that without this, people may be reluctant to share their personal or health information. Some justified their concerns using examples of the increasing number of scams and false information and the potential for insurance companies to gain access to their data and adjust premiums where there was higher risk.

### Threat Appraisal: Salience of Risk Information

Many participants appreciated the simplicity of the risk score presented in the “Relative Risk” prototype as it helped them understand “how far off they were from either end.” The first 2 iterations also included the risk of diabetes in the next 5 and 10 years, but these were “too far off” to be meaningful for some participants. Instead, they suggested reflecting a single score accompanied by a reminder that risk increases with age.

Reflecting on the term “Metabolic Age,” participants expressed concern that the tool may be intimidating and inaccessible, as it may not be clear how it relates to the risk of diabetes. However, upon probing, participants of all ages were able to explain the “metabolic age” concept and accurately perceive how it is related to one’s risk of diabetes. Participants anticipated the “Metabolic Age” prototype to motivate behavior change because of the urgency generated when observing their metabolic age to be older than their chronological age.

Both “Relative Risk” and “Metabolic Age” being relative to others or to their chronological age was noted to be helpful in answering the questions “Where am I?” and “Where should I be?” Participants joked about their desire to “beat (their) past self, and to beat others,” reflecting the local kiasu (fear of losing) mentality.

In the third iteration, both of these prototypes were shown to participants to assess which of the 2 risk presentations they preferred, and reactions were mixed. The familiarity of “Relative Risk” was perceived as the safe choice but had limitations in motivating behavior change, as it may be too “technical” and “boring.” In contrast, “Metabolic Age” required probing to be understood but was perceived as impactful and motivating for behavior change to help “[their] body to get younger.”

### Coping Appraisal: Facilitators for Behavior Change

On the interventions page, observing how the recommended behaviors can impact their risk of T2D was anticipated to be a powerful incentive to commit to change. The demonstrated impact of prospective behavior change on their risk scores was described as the “good news that followed the bad news.” Many participants referred to this as the most important part of the tool as it showed what one can do to reduce their risk of T2D and the potential magnitude of this change.

Feedback included the desire for personalization of the tool, with action items tailored to individual circumstances and focusing on areas where they were not “doing well enough.” For example, a participant shared that if their BMI is already low, they did not wish to see suggestions to eat a salad. Participants indicated that they would prefer the recommended behaviors to be small and specific steps that they could take in the context of their usual day-to-day lives.

Several participants mentioned that their interest in the risk communication tool would be sustained if these risk results could be integrated with health and exercise trackers, such as Fitbit or Apple Watch. There were suggestions to follow models of rewarding goals accomplished to enhance motivation, like the “National Steps Challenge” does with financial incentives.

## Discussion

### Principal Findings

We evaluated “Relative Risk,” “Metabolic Age,” and “Diabetes Onset” to assess which messaging would be the most effective risk communication tool. The predictive nature of “Diabetes Onset” was received poorly, likely due to its negative connotation. Future risk evaluating predictive framing and messaging could consider looking into shifting the concept into a positive frame like “T2D-free life-years” to assess if that is received differently. While “Relative Risk” was understood well due to its simplicity, “Metabolic Age” performed better in creating urgency to undertake preventative behavior. These 2 prototypes will require further testing to determine which prototype will be more effective in motivating uptake of T2D preventative behavior and inform future implementation.

Compared with the Heart Age [27] and Lung Age [28], the Metabolic Age refers to a complex process of metabolism instead of a single organ, which may require a greater level of health literacy to understand. This may explain why participants perceived the concept of the Metabolic Age as less accessible. In addition, the terms “metabolic age” and “metabolic risk” have been used in multiple contexts and allude to many diseases. If such a risk communication is implemented in the health system, appropriate education and awareness for the population will be necessary to avoid any misunderstandings.

The differing urgency to undertake preventative behavior could be explained by the present bias heuristic, where potential future benefits are undervalued [29]. The “Relative Risk” prototype presents a risk of developing diabetes in the next 10 years, whereas the “Metabolic Age” presents the current status of metabolic functioning. Hence, the salience of the current status of the body creates a more relatable feeling-at-risk and perceptions of immediate benefits to influence the urgency of engaging in the preventative behavior of T2D.

The intervention page is received as the most important part of the tool and is aligned with existing literature, where coping appraisal is the strongest predictor of subsequent behavior change [19]. The demonstration of potential improvement of their risk from preventative behavior was well-received and seen as the “good news following the bad news.” This perception can be explained by the preference of gain framing over loss framing in the context of disease prevention research [30]. The desire to improve and regain control of one’s health status could be a nod towards Self-Regulation Theory (SRT) [31,32]. A risk communication tool could act as an external stimulus or trigger to motivate intention for behavior. To support sustainable behavior change, it is likely that more comprehensive interventions enhancing key components of SRT, like goal-setting, self-monitoring, and self-efficacy, will be needed as a follow-up strategy [33].

Trust in the institution providing the tool was a crucial factor in influencing the way the prototypes were received. The desire for the risk assessment tool to be linked to official health records and hence protected securely by governmental institutions illustrates the need for the intervention to be embedded within the larger healthcare context. Following such implementation can provide an opportunity to leverage “pre-accumulated” interagency trust, enabling key players to coordinate the dissemination of information and interventions efficiently [34]. However, integrating with official health care records may limit access to only those who have access to health care. This element of trust also materialized in the credibility of the risk result, which influences the threat appraisal. Studies investigating risk communication during recent infectious disease outbreaks also found correlations between trust in the messenger and the public’s threat and coping appraisal, which impacted hygiene practices and physical distancing measures [35].

The digital delivery of such a tool will need to pay close attention to the user experience and concise messaging. As noted in the findings, the appeal and ease of use were often the first impressions from users. A high barrier to use could negate the impact of the tool regardless of how effective the risk framing may be. The difference in engagement with the digital prototypes versus the paper-based prototypes is likely due to the shortened attention span and quicker need for satisfaction arising from the current use of the internet and technology [36]. The length, format, and core message of the tool will need to grab the user’s attention quickly and make the message very easy to digest.

Throughout the development process, we adopted various participatory action research methods to design, test, and iterate for culturally sensitive and meaningful risk communication tools for T2D. The ideation workshops allowed us to codesign with participants based on specific insights, which allowed the evidence translation to be through a lay perspective, reducing assumptions and preconceived biases. It has been recognized that when developing interventions, researchers and clinicians may fail to include significant design and content elements or propose an incorrect design, especially when it comes to addressing the needs of minority groups [37].

However, often, those who are already health-seeking are the ones to participate in traditional health research or in efforts in which we ask participants to come to us. Hence, the PPI Hawker method allowed us to bring the research to the public and engage with those who may not be as health-seeking, which appropriately reflects the users who would benefit from such a tool. Guiding decision-making based on public opinion and working closely with our lay facilitators increases the potential impact and accessibility for users with different levels of health literacy and cultural traits [38,39].

The study’s strong focus on the Singaporean culture and values may limit its generalizability. However, with Singapore’s diverse and multi-ethnic population, findings from the study could be used within the larger Asian context. Further, the key characteristics of risk communication tools identified, as well as the process of codesign, can be transferable to similar developments and contexts.

In this study, the risk communication tool prototypes were prefilled with a dummy character’s risk profile. The reactions gathered were anticipated perceptions. Subsequent testing will benefit from having these prototypes programmed with the risk prediction model so that participants can enter their own health data and react more appropriately to personalized data. Participants can assess and experience their actual risk results for a more accurate assessment of the intention of behavior change. Further, gathering empirical evidence on the constructs of PMT as intended, namely threat and coping appraisals, in the different prototypes can provide a better understanding of how the theory translates to practice.

### Conclusion

In this study, we used ideation workshops with key stakeholders to develop potential risk communication tools, which address the gaps in threat and coping appraisals in regard to T2D risk and its preventative behaviors. We applied the “PPI Hawker” method to test and iterate on the prototypes. Participants were split between the “Relative Risk” and “Metabolic Age” prototypes as the preferred risk messaging. Further testing using functional tools will be conducted to accurately assess the efficacy of risk communication tools to influence the intention of positive behavior change. The insights on the design process and valued characteristics of a risk communication tool can inform the future development of such interventions.

## Abbreviations

HbA_1c_: hemoglobin A_1c_
PPI: Patient and Public Involvement
SRT: Self-Regulation Theory
T2D: type 2 diabetes
TRIRISK: Tripartite Model of Risk Perception

## Data Availability

The datasets used and/or analyzed during this study are available from the corresponding author upon reasonable request.
